# Health Communication on the Internet: Promoting Public Health and Exploring Disparities in the Generative AI Era

**DOI:** 10.2196/66032

**Published:** 2025-03-06

**Authors:** Jamal Uddin, Cheng Feng, Junfang Xu

**Affiliations:** 1 Communication Department Cornell University Ithaca, NY United States; 2 Vanke School of Public Health Tsinghua University Beijing China; 3 Institute for Healthy China Tsinghua University Beijing China; 4 Department of Pharmacy, Second Affiliated Hospital, School of Public health Zhejiang University School of Medicine Hangzhou China

**Keywords:** internet, generative AI, artificial intelligence, ChatGPT, health communication, health promotion, health disparity, health, communication, internet, AI, generative, tool, genAI, gratification theory, gratification, public health, inequity, disparity

## Abstract

Health communication and promotion on the internet have evolved over time, driven by the development of new technologies, including generative artificial intelligence (GenAI). These technological tools offer new opportunities for both the public and professionals. However, these advancements also pose risks of exacerbating health disparities. Limited research has focused on combining these health communication mediums, particularly those enabled by new technologies like GenAI, and their applications for health promotion and health disparities. Therefore, this viewpoint, adopting a conceptual approach, provides an updated overview of health communication mediums and their role in understanding health promotion and disparities in the GenAI era. Additionally, health promotion and health disparities associated with GenAI are briefly discussed through the lens of the Technology Acceptance Model 2, the uses and gratifications theory, and the knowledge gap hypothesis. This viewpoint discusses the limitations and barriers of previous internet-based communication mediums regarding real-time responses, personalized advice, and follow-up inquiries, highlighting the potential of new technology for public health promotion. It also discusses the health disparities caused by the limitations of GenAI, such as individuals’ inability to evaluate information, restricted access to services, and the lack of skill development. Overall, this study lays the groundwork for future research on how GenAI could be leveraged for public health promotion and how its challenges and barriers may exacerbate health inequities. It underscores the need for more empirical studies, as well as the importance of enhancing digital literacy and increasing access to technology for socially disadvantaged populations.

## Background

Health communication on the internet has continually evolved, driven by technological advancements [1-4]. Over time, the public, along with health researchers, practitioners, and organizations, have increasingly embraced these technologies for diverse health-related purposes. The emergence of generative artificial intelligence (GenAI) represents a revolutionary development in health communication and public health promotion. GenAI, a novel communication tool, mimics human responses during follow-up inquiries. Its conversational nature is particularly advantageous for health communication, enabling it to address various health inquiries, deliver customized health information, provide education, offer consultations, assist in disease diagnosis, simplify complex health reports, and translate health-related questions into multiple languages [5-7].

However, challenges such as accuracy, skill requirements, and access barriers persist with this technology [8-11]. GenAI operates on a large language model (LLM) built from publicly available data sources, including Google, Wikipedia, and GitHub [12]. As a result, the outputs generated by GenAI should be approached with caution to mitigate potential health risks. The quality of AI-generated results depends on the user’s ability to develop effective prompts, input accurate text for inquiries, and access advanced features through subscriptions. Consequently, individuals with limited health literacy, insufficient prompt development skills, or an inability to afford premium subscriptions may miss out on these technological benefits, potentially exacerbating health disparities.

Earlier studies have examined the internet as a medium for health communication across various contexts, including persuasive health messaging via the World Wide Web [1], web-based consumer health information [13], the role of Web 2.0 in health and education [2], the internet’s role in health interventions [14], the use of social media for health promotion and behavior change [15], and the integration of mobile devices in health care and interventions [16]. Additionally, a comprehensive review of social media apps and their purposes has been conducted [17]. However, recent technological advances, such as wearable devices, the Internet of Things, blockchain, and GenAI, present new opportunities as health communication mediums. Research combining these latest technologies as health communication tools and examining their applications for health promotion and understanding health disparities remains limited.

To address this research gap, this viewpoint adopts a conceptual approach by integrating existing literature and targeted web searches [4,13,18] to provide an updated overview of health communication mediums and their role in understanding health promotion and disparities in the GenAI era. Additionally, health promotion and health disparities are briefly analyzed through the lenses of the Technology Acceptance Model 2 (TAM2) [19], the uses and gratifications theory [20], and the knowledge gap hypothesis [21].

This conceptual study is organized into three sections as follows: (1) a brief overview of internet-based communication mediums and their health promotion practices, (2) the digital divide on the internet, and (3) health promotion and health disparities in the era of GenAI.

### Health Communication Mediums on the Internet

The internet has become a vital medium for health communication in the era of Web 2.0 [22], evolving across various domains such as social media [17,23], mobile health [24], the Internet of Things [25], blockchain [26], wearable devices [27,28], and, more recently, GenAI [12]. These mediums also include health websites [13], health forums and communities [29], health-related webinars and podcasts [30,31], telemedicine [32,33], electronic health records [34], voice assistants [35,36], and augmented and virtual realities [37], all of which support a wide variety of health promotion practices and research [13].

Internet-based resources offer numerous features for persuasive communication aimed at promoting health behavior [1]. Therefore, an updated list of these communication mediums and their potential for health promotion practices are presented in Table 1, serving as a valuable resource from a health communication perspective.

**Table 1:** Major health communication mediums and their potential for health promotion practices.

Web-based health communication mediums	Potentiality	Health promotion practices (examples)
Web 2.0	Interactivity (eg, chat rooms, emails, and hyperlinks)	Responsive user interface; provision of rich content including text, images, and videos; and reusability and collaboration opportunities
Health websites	Featured health content (eg, health articles, audios, videos, images, and infographics)	Mayo Clinic, WebMD, and Centers for Disease Control and Prevention
Social media	Networking (eg, social support, community development, knowledge sharing, and doctor-patient interactions)	X, Facebook, WeChat, YouTube, WhatsApp, and Instagram
Health forums and web-based communities	Groups with common health interests (eg, mutual support, discussion of common problems, patient empowerment, anonymity, and emotional support)	ParkinsonNet, COPD.com, and PatientLikeMe
Health-related webinars and podcasts	Health knowledge dissemination through live sessions or on demand (eg, narrative communication and health education)	Cancer Council Victoria, Hidden Brain, The Blindboy Podcast, and The Two Norries
Mobile health	Easy and on-the-go access to information (eg, web browsing, audio and video calls, and text messaging)	Cellphones, smartphones, and smartphones with specific software or apps
Telemedicine	Health services provided remotely (eg, using telecommunication technology for doctor-patient communication)	Physicians, nurses, and other allied health professionals
Electronic health records	Patient health records stored in computer hard drives or web-based servers (eg, easy access from anywhere for multiple parties, and easy for health decisions and diagnosis)	Physicians, insurance companies, and patients
Blockchain	Digital ledger (eg, secured data storing and exchange platform)	Health care industry and electronic health record researchers
Voice assistants	Conversational assistance (eg, health information inquiry with voice)	Amazon Alexa, Google Home, Google Assistant, Apple Siri, and Samsung Bixby
Wearable devices	Health monitoring and remote health services (eg, glasses, smartwatches, and suits)	Emergency medical service providers, family members, patients, public, and clinicians
Augmented reality and virtual reality	Enhanced views combining real and digital objects (eg, health education, training, and assessment)	Health care industry
Internet of Things	Connectivity and automation (eg, collecting, sending, receiving, storing, and measuring data of anyone, anything, anytime, any service, in any network)	Health care and health management
Generative artificial intelligence	Humanlike interaction digitally (eg, content generation regarding text, image, audio, video, synthetic data, and code)	Public, health care industry, health professionals, and researchers

### Digital Divide on the Internet

The opportunities for web-based health communication also contribute to the digital divide. The internet serves as a vast resource of health benefits for many who seek health information and use it for health purposes, depending on their access, skills, usage, and participation [38,39]. Educated, older, and tech-savvy individuals benefit the most from the internet for health-related purposes [38]. However, health content contributed by individuals without professional health knowledge or training creates complexities in finding reliable information. This can harm those who lack the ability to evaluate the credibility of information or its sources, as much of the web-based health content available is incomplete, misleading, or difficult to understand [39].

Today, people encounter misinformation in various ways, such as through search engines (eg, random searches on Google and Yahoo suggesting links based on top-matching results), visiting specific health websites (eg, the quality of information depends on whether the person is visiting a reputable site like WebMD), relying on user-generated content from social media or other websites (eg, Facebook, Twitter, Wikipedia, and Yelp), and using mobile apps that may not adhere to established medical guidelines [40]. Certain health topics are particularly susceptible to misinformation on social media, including vaccines, drugs and smoking, noncommunicable diseases, pandemics, eating disorders, and medical treatments [41].

Traditionally, the digital divide has been understood in terms of physical access to the internet, which depends on factors such as age, education, income, perceived health, and social isolation [42,43]. However, the digital divide for health purposes has widened due to low levels of health literacy (eg, the ability to understand, appraise, and use web-based information for health purposes) and inadequate internet use skills (eg, browsing and content-related skills), which are more prevalent among hard-to-reach communities. Non-English speakers, who make up around 13% of the US immigrant population, are less likely to use the internet for health purposes [44]. For example, the use of telemedicine for primary care visits by patients with limited English proficiency and digital access decreased proportionately in New York City during COVID-19 [45]. Such digital divides may further worsen during new waves of technological advancement, such as GenAI.

## GenAI: Health Promotion and Health Disparity

### Overview

GenAI presents a significant opportunity for health promotion and may have a revolutionary impact on the health care system by leveraging vast amounts of medical data and knowledge to support medical decision-making, patient communication, health education, and data management [7]. However, it may also contribute to health disparities due to limited access, insufficient prompt development skills, and low health literacy among many populations [44].

This section provides an overview of the major GenAI channels, their potential for health promotion, and how their limitations, particularly lack of access and skills, may exacerbate health disparities.

### GenAI and Health Promotion

#### Application of GenAI

GenAI is poised to transform content creation across text, image, audio, and video formats [46]. Its ability to generate humanlike responses based on prompts has created new opportunities for health promotion. Potential users of these tools include the general public, health researchers, practitioners, and health organizations [5-7,17].

A variety of GenAI tools are currently available, each with a specific focus, such as text generation, and audio, video, and image creation [5,9,46-50]. Table 2 provides a brief overview of some notable GenAI channels, their potential uses, target users, and the skill requirements for health-related purposes.

**Table 2:** Generative artificial intelligence (GenAI) channels and their potential use, users, and skills requirement for health purposes.

GenAI channels		Use	Users	Skills
**Text generation**				
	ChatGPT	Humanlike conversation (eg, personalized health information, answering health-related questions, interaction with chatbots, and facilitating patient-provider communication)	Individuals, health educators, caregivers, and health organizations	Prompt development skills (eg, knowledge of medical terminology, patient communication principles, and using empathy in responses)
	Gemini	Visual content creation from textual descriptions (eg, health literacy and health education)	Individuals, health care educators, health organizations, medical illustrators, and health communicators	Prompt development skills, medical concepts, and visual communication techniques
	Microsoft Copilot	Development of health-related software apps and digital tools	Health IT professionals, software developers, and digital solution providers	Prompt development skills, knowledge of health care standards, Health Insurance Portability and Accountability Act compliance, and user interface and user experience design principles
	Jasper	High-quality, text-based content generation (eg, blog posts and social media posts)	Content creators, patient educators, and health organizations	Prompt development skills, understanding health topics, medical knowledge, and ethical considerations
**Image, audio, and video generation**				
	DALL-E 3 and Midjourney	Concept art, illustration, and graphic design	Artists, designers, and concept artists	Basic prompt development skills and familiarity with different artistic styles
	JukeBox and MuseNet	Music composition, songwriting, and sound design	Musicians, producers, and game and film developers	Text prompt development and musical knowledge
	Synthesia and Runway	Educational videos, marketing and communication, and social media content development	Educators, marketers, communicators, and content creators	Prompt development, basic video editing skills, and understanding the target audiences

#### Health Promotion Through GenAI

Traditionally, individuals communicated with health care professionals, family members, and friends for health-related purposes [51,52]. This communication provided immediate responses to health needs and allowed for follow-up questions. However, barriers included difficulties in reaching people in real time, time and cost constraints, limited understanding of health issues, and discomfort in discussing stigmatized health topics like addiction and sexually transmitted infections. The development of Web 2.0, social media, and mobile apps addressed many of these barriers [53-55]. These platforms offer health information from both individuals and professionals, enabling people to access information as needed, provided they possess the necessary technology, internet skills, and the ability to process web-based content [38,39]**.**

Despite these advancements, existing mediums have limitations, such as the lack of interactive health information without human involvement, delayed responses, limited data for mobile app responses, difficulty understanding health information in specific contexts, and limited accessibility for non-English speakers. GenAI has the potential to overcome these barriers by providing interactive, real-time responses and personalized health information based on LLMs. GenAI’s ability to pass challenging exams (eg, the US Medical Licensing Exam and the USA Biology Olympiad Semi-Final Exam) underscores its capacity to deliver tailored responses aligned with human needs and inquiries [56]**.**

Individuals can now fulfill various needs—such as health inquiries, image development, and health content creation—using GenAI tools, if they have access to these tools and effective prompt writing skills. The acceptance and use of this new technology can also be explored through theoretical frameworks.

#### Theoretical Application of GenAI

The application of GenAI can be examined through the TAM2 and the uses and gratifications theory. TAM2 suggests that an individual’s acceptance or rejection of a system or technology depends on its perceived usefulness and ease of use [19]. Perceived usefulness is determined by factors such as the quality of the outcomes and the benefits provided, while perceived ease of use refers to how simple the technology is to operate, learn, and become proficient with, requiring minimal effort [57]. GenAI has demonstrated its usefulness by solving complex problems, generating humanlike responses to diverse inquiries, creating content, and acting as an assistant. As a result, health educators, medical professionals, and researchers have started incorporating these tools into their work. Consequently, the general public is increasingly likely to adopt GenAI for health purposes, such as obtaining personalized health information, interpreting health reports, and making informed health decisions.

The technology is also designed to be user-friendly, resembling the simplicity of a Google search. Major GenAI tools, such as ChatGPT, Gemini, and Copilot, are accessible with straightforward sign-up processes and offer basic services at no cost. The rapid adoption of this technology is evident from its widespread use. For example, since its launch on November 30, 2022, ChatGPT has amassed over 300 million users weekly, with 1 billion messages sent by its users on the platform each day [58]. In the United States, ChatGPT app downloads exceeded 32.6 million, and Gemini, launched on December 6, 2023, reached 12.67 million downloads by September 2024 [59]. However, realizing the full benefits of this technology often depends on the user’s prompt development skills.

The motives behind the widespread use of GenAI can be analyzed through the uses and gratifications theory [20]. According to this theory, individuals consciously select specific media to fulfill their needs. The primary motivations for using GenAI include its humanlike responsiveness, which addresses limitations in conventional internet-based communication; its ability to deliver tailored health information, unlike standard search engines that primarily return links; and its capacity to meet personalized health needs, such as seeking health information, diagnosing conditions, making health decisions, and creating health-related content. Additionally, the ability to use AI agent services for task completion motivates individuals, particularly health researchers, campaign designers, educators, and medical professionals, to integrate GenAI into their work.

Despite its significant potential, GenAI has limitations that may contribute to health disparities.

### GenAI and Health Disparity

#### Overview

GenAI has several limitations, including its reliance on specific databases, difficulty in understanding users’ unique contexts, and individuals’ inability to fully use its benefits. Some of these limitations are discussed below:

#### Limitations of GenAI

GenAI relies on LLMs trained on data from sources such as Google, Wikipedia, and GitHub. However, because data on the internet can include inaccurate information and misinformation—often contributed by individuals without relevant expertise or oversight—LLM-based AI tools may generate inaccurate or biased responses. For example, researchers evaluated ChatGPT-4’s health-related information in the field of radiology and found that only 65% of the information was accurate [11]. Similarly, while the same tool passed the medical licensing exam, it achieved a passing mark of approximately 60% [56], suggesting a potential inaccuracy or bias rate of over 30%.

#### Limitations of Personalized Health Advice

Personalized health care relies on various factors specific to an individual’s health condition, typically assessed through diagnostic methods such as pulse checks, blood pressure readings, blood tests, x-rays, and cultural considerations. While GenAI tools can provide health suggestions based on detailed prompts or text inputs, their recommendations are derived from universal datasets, which may not always be applicable to personalized care [9]. Furthermore, GenAI has limitations in understanding individual cultural and racial contexts [60], which are critical for determining health care needs and preferences. As a result, researchers recommend verifying GenAI-generated outputs with qualified health care professionals to ensure accuracy and relevance.

#### Individual Incapacity to Evaluate Health Information

Health literacy, the ability to assess information accuracy, and knowledge of reliable sources are essential for individuals to evaluate specific health information and use it effectively [61]. Individuals who possess these skills can maximize the benefits of GenAI technology. However, racial and ethnic minority groups, who often experience lower levels of health literacy and lack the skills needed to evaluate health information, may be disproportionately deprived of these technological advantages.

#### Limitations of Access to GenAI Tools

Although 95% of American adults have internet access [62], many individuals with lower health literacy, language barriers, or limited financial resources face challenges in accessing the full benefits of GenAI tools. These tools often require paid subscriptions to unlock advanced features and enhanced accuracy. For instance, ChatGPT Plus, a subscription-based version, delivers more accurate outputs compared with the free version, ChatGPT 3.5 [10].

#### Lack of Skills in Prompt Development

The quality of GenAI output is highly dependent on effective prompt development, which involves crafting precise text inputs for the tools [8]. Individuals proficient in prompt development—using techniques such as open-ended questions and using relevant keywords—can maximize the benefits of this technology. Conversely, individuals with lower levels of education, poor health literacy, or limited technological proficiency may struggle to harness its potential benefits, leaving them at a disadvantage.

### Potential Health Disparity Through GenAI

Health disparity refers to differences in health status between disadvantaged social groups—primarily individuals with low income, racial and ethnic minority groups, women, and others who consistently experience worse health and greater health risks compared with advantaged social groups [63]. In the context of web-based health benefits, health disparity is influenced by limitations in access to information technology, poor health literacy, language barriers, and inadequate internet use skills, such as browsing and content-related skills [44].

In the era of GenAI, health disparities are likely to persist among these groups due to challenges in evaluating GenAI outcomes and difficulties in prompt development. Current ChatGPT users, predominantly college students (43%) and Fortune 500 companies (80%), are likely drawn from higher social groups that benefit most from this technology. These advantaged groups, with higher levels of education, communication skills, and acceptance of new technology, are more likely to maintain superior health knowledge and better health outcomes compared with disadvantaged groups, consistent with the knowledge gap hypothesis [21]. This theory posits that individuals with higher social status acquire information faster than those with lower social status. Consequently, health disparities are compounded as higher-status individuals use new technologies more effectively for purposeful activities, while less-educated individuals often use technology primarily for entertainment [64].

To reduce these disparities, efforts must focus not only on the availability of technology but also on improving accessibility, enhancing digital literacy, fostering engagement, and building trust [3].

## Conclusion

This study provides an overview of major health communication mediums on the internet, their practices, and the limitations of traditional web-based health communication tools for health purposes. It highlights GenAI as a new avenue for health promotion while addressing its potential to exacerbate health disparities. These discussions offer valuable insights for researchers, educators, and health professionals focusing on internet-based health communication and health promotion practices.

Although this research lacks extensive evidence-based claims, it serves as a foundation for further empirical studies, such as interviews and surveys with marginalized communities, to better understand health disparities in the era of GenAI. The study could be further enhanced by conducting a systematic review to provide a more comprehensive understanding of communication mediums, their health-related practices on the internet, and health disparities in the rapidly evolving context of GenAI.

Moreover, policy makers and health educators should implement targeted programs to familiarize disadvantaged groups with the latest technologies and their benefits. Efforts should focus on reducing health disparities by improving digital literacy, enhancing prompt development skills, and increasing access to technology for underserved populations.

## Abbreviations

AI: artificial intelligence
GenAI: generative artificial intelligence
LLM: large language model
TAM2: Technology Acceptance Model 2

## Data Availability

All data generated or analyzed during this study are included in this published paper.
